# Enhanced Structural Control of Soft-Templated Mesoporous Inorganic Thin Films by Inert Processing Conditions

**DOI:** 10.1021/acsami.2c18090

**Published:** 2022-12-12

**Authors:** Maximiliano Jesus Jara Fornerod, Alberto Alvarez-Fernandez, Eric R. Williams, Maximilian W. A. Skoda, Beatriz Prieto-Simon, Nicolas H. Voelcker, Morgan Stefik, Marc-Olivier Coppens, Stefan Guldin

**Affiliations:** † Department of Chemical Engineering, University College London, Torrington Place, London WC1E 7JE, U.K.; ‡ Department of Chemistry and Biochemistry, University of South Carolina, Columbia, South Carolina 29208, United States; § ISIS Pulsed Neutron and Muon Source, Rutherford Appleton Laboratory, Harwell, Oxfordshire OX11 OQX, U.K.; ∥ Department of Electronic Engineering, Universitat Rovira i Virgili, 43007 Tarragona, Spain; ⊥ ICREA, Pg. Lluís Companys 23, 08010 Barcelona, Spain; # Monash Institute of Pharmaceutical Sciences, Monash University, Parkville, Victoria 3052, Australia; ∇ Melbourne Centre for Nanofabrication, Victorian Node of the Australian National Fabrication Facility, Clayton, Victoria 3168, Australia; ○ Centre for Nature Inspired Engineering, University College London, Torrington Place, London WC1E 7JE, U.K.

**Keywords:** thin films, mesoporous, calcination, sol−gel, block copolymers

## Abstract

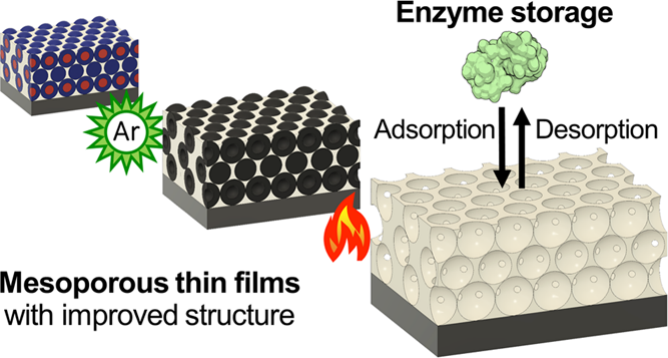

Mesoporous thin films are widely used for applications in need of high surface area and efficient mass and charge transport properties. A well-established fabrication process involves the supramolecular assembly of organic molecules (e.g., block copolymers and surfactants) with inorganic materials obtained by sol–gel chemistry. Typically, subsequent calcination in air removes the organic template and reveals the porous inorganic network. A significant challenge for such coatings is the anisotropic shrinkage due to the volume contraction related to solvent evaporation, inorganic condensation, and template removal, affecting the final porosity as well as pore shape, size, arrangement, and accessibility. Here, we show that a two-step calcination process, composed of high-temperature treatment in argon followed by air calcination, is an effective fabrication strategy to reduce film contraction and enhance structural control of mesoporous thin films. Crucially, the formation of a transient carbonaceous scaffold enables the inorganic matrix to fully condense before template removal. The resulting mesoporous films retain a higher porosity as well as bigger pores with extended porous order. Such films present favorable characteristics for mass transport of large molecules. This is demonstrated for lysozyme adsorption into the mesoporous thin films as an example of enzyme storage.

## Introduction

Inorganic mesoporous thin films are ideally suited for applications requiring high surface area and good mass and electron transport properties, including gas sensors,^1^ fuel cells,^2^ Bragg reflectors,^3^ permselective membranes,^4^ dye-sensitized solar cells,^5^ batteries,^6^ supercapacitors,^7^ enzyme storage,^8^ electrochemical biosensing,^9^ and catalysis.^10^ In most cases, the interplay between porosity and pore size is crucial for their operation. However, most existing fabrication processes lack fine control over these characteristics, constraining functionality for material demands beyond a high surface area.

One of the most attractive approaches to fabricate inorganic mesoporous thin films relies on the supramolecular assembly of sol–gel precursors with organic molecules that self-assemble on the nanoscale.^11−13^ The use of amphiphilic block copolymers (BCP) as soft templates offers distinct advantages. For example, BCPs provide access to the entire mesopore size range (between 2 and 50 nm), diverse pore morphology, and various porosities by varying the length, ratio, and number of polymeric blocks.^14−19^ On the other hand, sol–gel chemistry is a versatile synthesis method that offers access to a comprehensive library of materials, including transition-metal oxides, ceramics, and glasses (e.g., aluminosilicates, Al_2_O_3_, SiO_2_, TiO_2_, indium tin oxide, Nb_2_O_5_).^20,21^

Thin film processing typically consists of three steps.^22^ First, the BCP host and the inorganic guest materials are coassembled in the so-called hybrid solution. For example, in this work, we mixed the amphiphilic diblock copolymer poly(isoprene)-*block*-poly(ethylene oxide) (PI-*b*-PEO) and aluminosilicate nanoparticles in an azeotropic mixture of 1-butanol and toluene. In this step, the hydrophobic interactions of the PI block with the solvent induce the BCP to self-assemble into micelles. Simultaneously, the hydrophilic PEO block forms hydrogen bonds with the nanoparticles. Second, the solution is dispersed onto a substrate, generating a hybrid thin film. Third, the inorganic nanoparticles condense into an aluminosilicate network, followed by the removal of PI-*b*-PEO to reveal the inorganic porous structure.

Calcination in air is often preferred over other methods (such as oxygen plasma, solvent extraction, and UV–ozone treatment) to remove the polymer from the inorganic matrix for its dual effect on the film. First, temperature induces the condensation of the inorganic component, generating a continuous solid network. Second, the oxidizing conditions degrade the BCP to reveal the pores.^23^ However, this process also has a detrimental effect on the films. Several studies have shown that the hybrid layer undergoes uniaxial contraction of up to 70% in the direction perpendicular to the substrate, resulting in flattened pores with limited access.^24−31^ This anisotropic shrinkage is often attributed to the residual solvent evaporation and mass loss of the sol–gel-derived network during the condensation reaction,^27,32−34^ combined with a film firmly attached to the substrate.^35^ Consequently, the thin film densifies during calcination, with contraction of both porosity and film thickness, which limits in particular mass-transport-dependent applications.

Processing methods to reduce the uniaxial film shrinkage of inorganic materials are limited to date. For instance, sequential deposition and annealing in a layer-by-layer approach allows to alleviate crack formation by inducing film shrinkage during gradual layer build-up.^34^ This protocol permitted a simple and fast fabrication of thicker mesoporous TiO_2_ films for solar cell applications. Another processing protocol requires pretreating the hybrid film with liquid paraffin to stimulate the condensation of the sol–gel precursors before calcination, thus reducing the uniaxial contraction of mesoporous TiO_2_.^36^ The use of microwave-assisted template removal showed to reduce shrinkage of siliceous porous materials due to the fact that the rapid and localized heat condensed the sol–gel while removing the organics.^37^

In this paper, we apply a simple two-step calcination process, namely, high-temperature treatment in argon, followed by air calcination, to reduce the unfavorable effects of direct thermal combustion of the templating agent on inorganic mesoporous thin films, here demonstrated for aluminosilicates. In the first step, high temperature coupled with an inert atmosphere carbonizes the BCP, creating a scaffold that keeps the inorganic matrix in place during the condensation reaction. In the second step, calcination in air removes the carbon scaffold from the inorganic network to reveal the pores. Methods using an inert atmosphere to improve the properties of bulk mesoporous inorganic materials have been previously reported. For instance, Lee et al. introduced a method referred to as combination of assemblies by soft and hard chemistries (CASH), which enabled the formation of solid crystalline wall structures at temperatures that would otherwise lead to the destruction of the pores.^38^ However, as we show in this work, the impact of calcination on inorganic thin films is fundamentally different to that of bulk materials, which shrink isotropically upon condensation.^35^ To the best of our knowledge, the effect of inert processing conditions on the structural preservation of mesoporous thin films has not been investigated to date. We apply a range of analytical tools, including spectroscopic ellipsometry (SE), ellipsometric porosimetry (EP), Fourier transform infrared (FTIR) spectroscopy, grazing-incidence small-angle scattering (GISAXS), and atomic force microscopy (AFM) to track structural parameters at processing steps and identify an ideal experimental procedure to exploit transient carbon scaffolding for improved structural control in mesoporous films with thickness up to 500 nm after calcination. Finally, we use a quartz crystal microbalance (QCM) to demonstrate the benefits of using this two-step processing protocol for the mass transport of large molecules.

## Results and Discussion

### Two-Step Calcination Protocol

**Figure 1:**
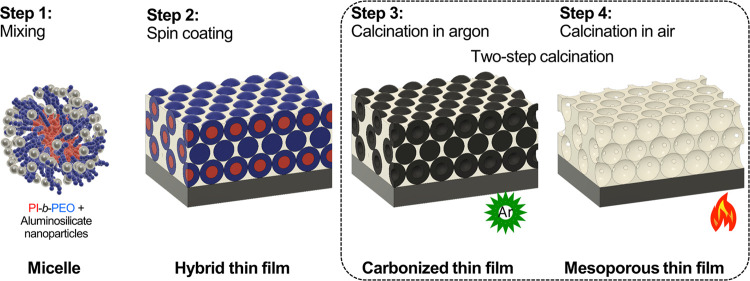
Schematic of the mesoporous thin film fabrication process via two-step calcination.

Hybrid thin films were fabricated following a protocol reported elsewhere.^14,15^ In brief, a previously hydrolyzed aluminosilicate sol from aluminum tri-sec-butoxide and (3-glycidyloxypropyl)-trimethoxysilane (GLYMO) were mixed with the structure-directing agent PI-*b*-PEO prior to thin film deposition by spin-coating. The hybrid films were subsequently annealed in argon at a temperature of 450 °C. Under such inert conditions, the thermally stable sp^2^-hybridized carbon of the hydrophobic polymeric PI block is expected to convert into a carbonaceous residue.^38^ Subsequently, air calcination at 450 °C was applied to remove the carbon scaffold of the film and fully expose the mesoporous architecture. An overview of the fabrication process involving “two-step” calcination is shown in Figure 1. A schematic indicating the main structural parameters of a mesoporous thin film discussed in this work is provided in Figure S1.

### Calcination of Mesoporous Thin Films: Oxidizing vs Inert Conditions

**Figure 2:**
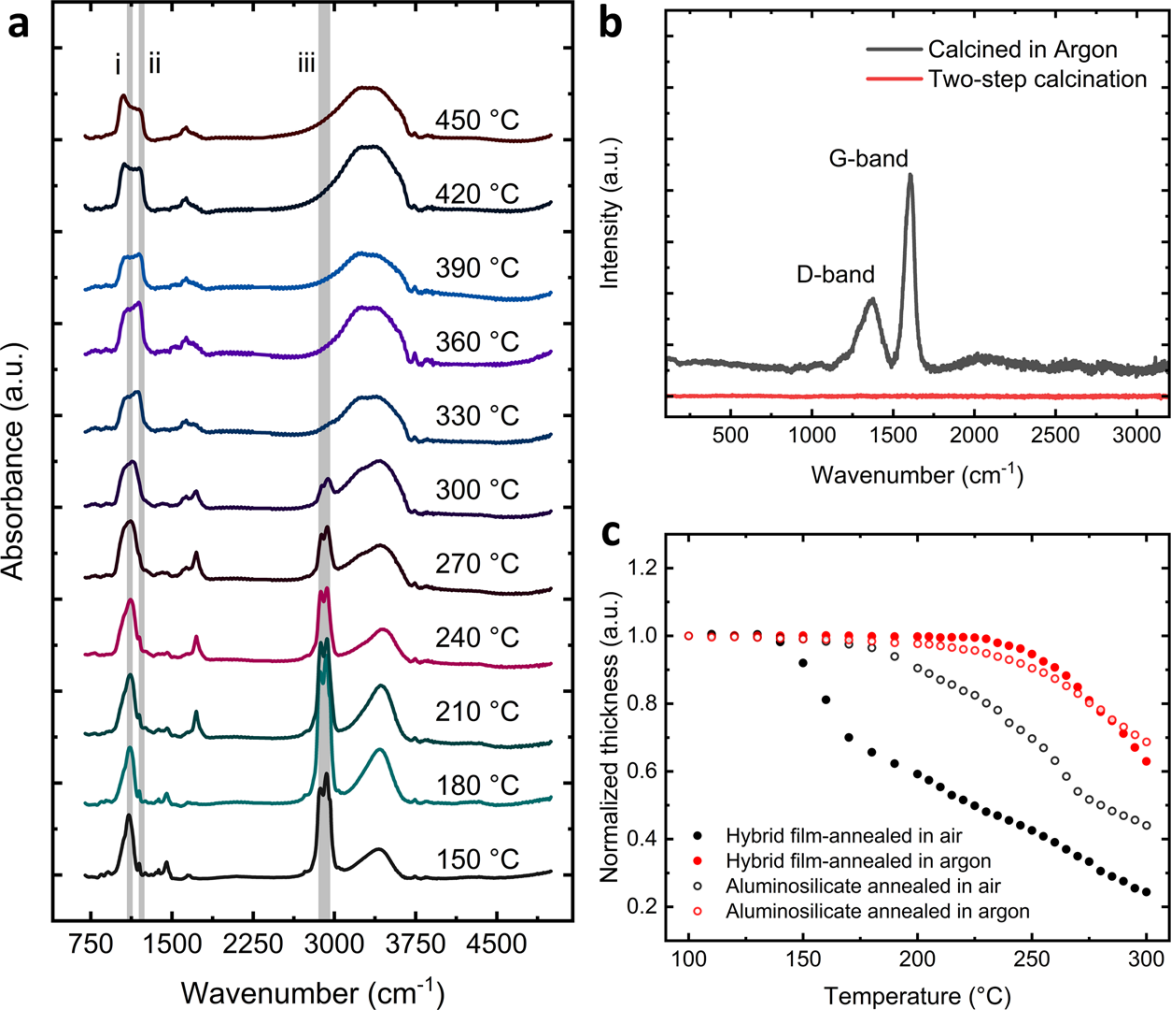
Hybrid thin films annealed at different temperatures. (a) FTIR spectra of hybrid films annealed from 150 to 450 °C in air. (b) Raman spectra of a carbonized hybrid film after annealing in argon at 450 °C, showing the characteristic carbon bands (D-band and G-band) and the spectra of the same film after air calcination, showing the disappearance of the carbon bands. (c) *In situ* ellipsometric measurements showing the film thickness evolution of samples annealed in air and argon.

A disadvantage of calcination procedures in air is the significant uniaxial contraction or thickness shrinkage.^24−26^ In addition to the initial solvent evaporation, a well-known source of contraction in sol–gel chemistry is the mass loss due to the underlying condensation reaction.^34^ In silicates, temperature induces the condensation of silanol (Si–O–H) groups on sol particles into a continuous siloxane (Si–O–Si) network, generating water and alcohol molecules in the process.^39^ This results in the shrinkage of the material along the silicate-rich directions, with an anisotropic effect on thin films because they are attached to the substrate.^35^ To understand the effect of temperature on the kinetics of the concurrent processes observed under oxidizing conditions, i.e., aluminosilicate sol–gel reaction and organic thermal combustion, we employed Fourier Transform Infrared (FTIR) spectroscopy. In Figure 2a, the respective spectra of samples annealed at temperatures ranging from 150 to 450 °C in air atmosphere are shown. We used the Si–O (1120 cm^–1^) and Al–O–Si (1220 cm^–1^), and C–H (2880 and 2950 cm^–1^) stretching bands to track the condensation reaction and organic degradation, respectively (Figure 2a gray bands i, ii, and iii). The Si–O and Al–O–Si stretching bands correspond to the inorganic network. The C–H stretching bands are the fingerprint of the organic content, in this case, PI-*b*-PEO and residual organic molecules from the sol–gel hydrolysis. The FTIR spectrum of the sample calcined in air at 450 °C served as a reference sample, where the aluminosilicate structure is expected to be fully condensed, and the PI-*b*-PEO degraded. We found that the C–H stretching band decreased in intensity at 240 °C and totally disappeared between 300 and 330 °C, indicating BCP degradation. In contrast, we observed that the inorganic network continued to evolve beyond 330 °C, as evidenced by the broadening of the Si–O stretching band. Also, a new peak emerged between 270 and 300 °C, which corresponds to the Al–O–Si stretching band, indicating that aluminium starts entering the siloxane network.^39^ Please note that we did not observe changes in the Si–O stretching band at temperatures below 180 °C, which is typically referenced in the literature as a suitable annealing temperature.^15,40,41^ In consequence, we did not apply intermediate annealing procedures but calcined the thin films directly after spin-coating.

FTIR measurements showed that the complete degradation of PI-*b*-PEO occurred between 300 and 330 °C in samples calcined in air, while the condensation of the inorganics started at 180 °C and continued up to 450 °C. Therefore, a significant portion of condensation, i.e. from 300 to 450 °C, occurred without any structural support provided by the BCP. In contrast, calcination in argon promoted the *in situ* formation of carbon species that remained in the film at this temperature range, as depicted by the peaks of the disordered (D-band, 1350 cm^–1^) and graphitic (G-band, 1600 cm^–1^) carbon bands in the Raman spectra shown in Figure 2b.^42^ Please note that previous studies have shown that calcination in air may not remove all carbon residues. While Raman spectroscopy is reliable in detecting carbon bonds elastic backscattering spectrometry (EBS) would be required to identify atomic carbon traces (which could be removed by oxygen plasma etching).^23^

To understand the contribution of the carbonization step to reduce the uniaxial contraction of the mesoporous film, we studied *in situ* the thickness evolution upon heating of films fabricated with 50 wt % BCP using a spectroscopic ellipsometric setup with a temperature and atmosphere-controlled chamber. We found that the thickness reduction of hybrid films annealed in argon was significantly delayed when compared with films directly annealed in air, as shown in Figure 2c. While at 250 °C in argon atmosphere, the hybrid films still exhibited 99% of their initial film thickness, this was already reduced to 40% in air. At 300 °C, the difference compared to the initial thickness pre-processing was found to be 70% vs 25%. We attributed the higher film shrinkage observed in films annealed in air up to 300 °C to the premature degradation of the BCP. We also noticed that the thickness reduction for hybrid films annealed in air (Figure 2c, black dots) occurred earlier than pure aluminosilicate films annealed in air (Figure 2c, black circles). This suggests that the BCP acted mainly as a porogen under oxidizing conditions rather than to provide structural support. In contrast, annealing in argon led to prolonged retention of the initial film thickness in a temperature range relevant for the build-up of the inorganic network, thus contributing to the structural integrity of the films.

### Effect of Two-Step Calcination on the Mesoporous Thin Film Structure

**Figure 3:**
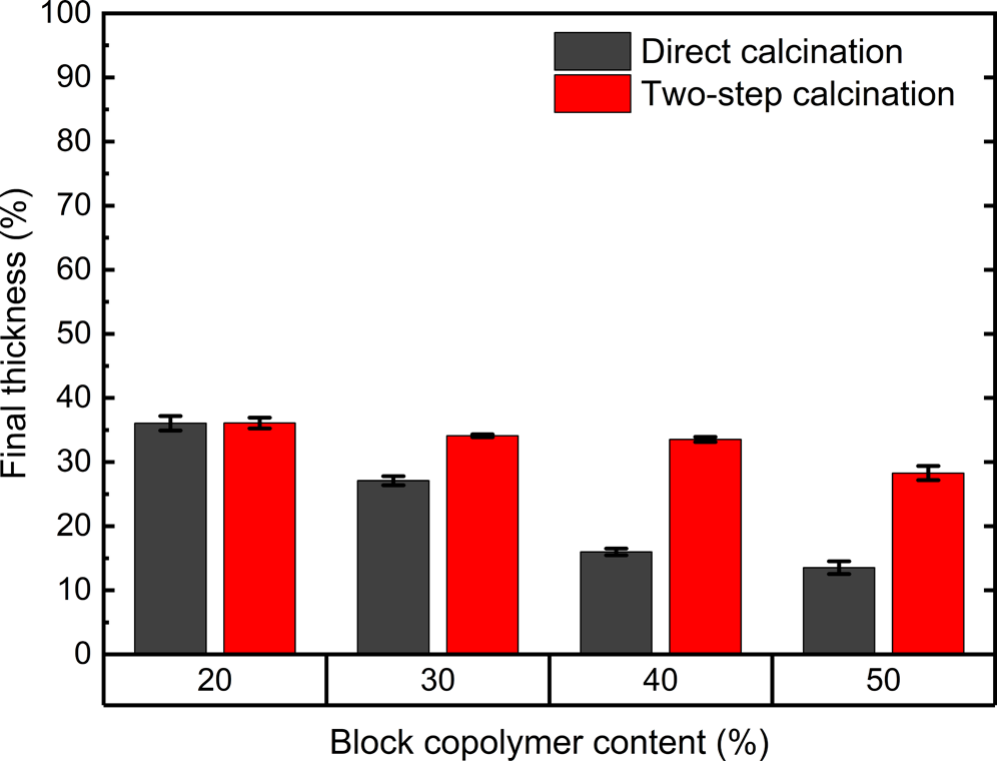
Film thickness after direct calcination (black bars) and two-step calcination (red bars) of aluminosilicate mesoporous films fabricated with different amounts of the block copolymer PI-*b*-PEO in hybrid solution.

To characterize the effect of inert processing conditions on the uniaxial film shrinkage, we fabricated mesoporous films with 20, 30, 40, and 50 wt % BCP in the mixture before spin-coating, labeled BCP_20_, BCP_30_, BCP_40_, and BCP_50_, respectively. We measured the film thickness before and after calcination by spectroscopic ellipsometry (SE). Figure 3 provides a graphical summary of the film thickness obtained after calcination as a percentage of the initial thickness. The uniaxial contraction was calculated as the ratio of the thickness after the final calcination step over the thickness in the hybrid state at room temperature. We found that the shrinkage for the samples BCP_20_, BCP_30_, BCP_40_, and BCP_50_ increased proportionally to the BCP content in films directly calcined in air, i.e., 64 ± 1, 73 ± 0.7, 84 ± 0.5, and 86 ± 0.9%, respectively. In contrast, the two-step processed films of similar BCP content underwent uniaxial contraction of 64 ± 0.8, 66 ± 0.8, 66 ± 0.4, and 72 ± 1%, respectively. The contrast in shrinkage leading up to a doubling of the retained film thickness provides an indication of the impact that early BCP degradation has on the uniaxial contraction.

**Figure 4:**
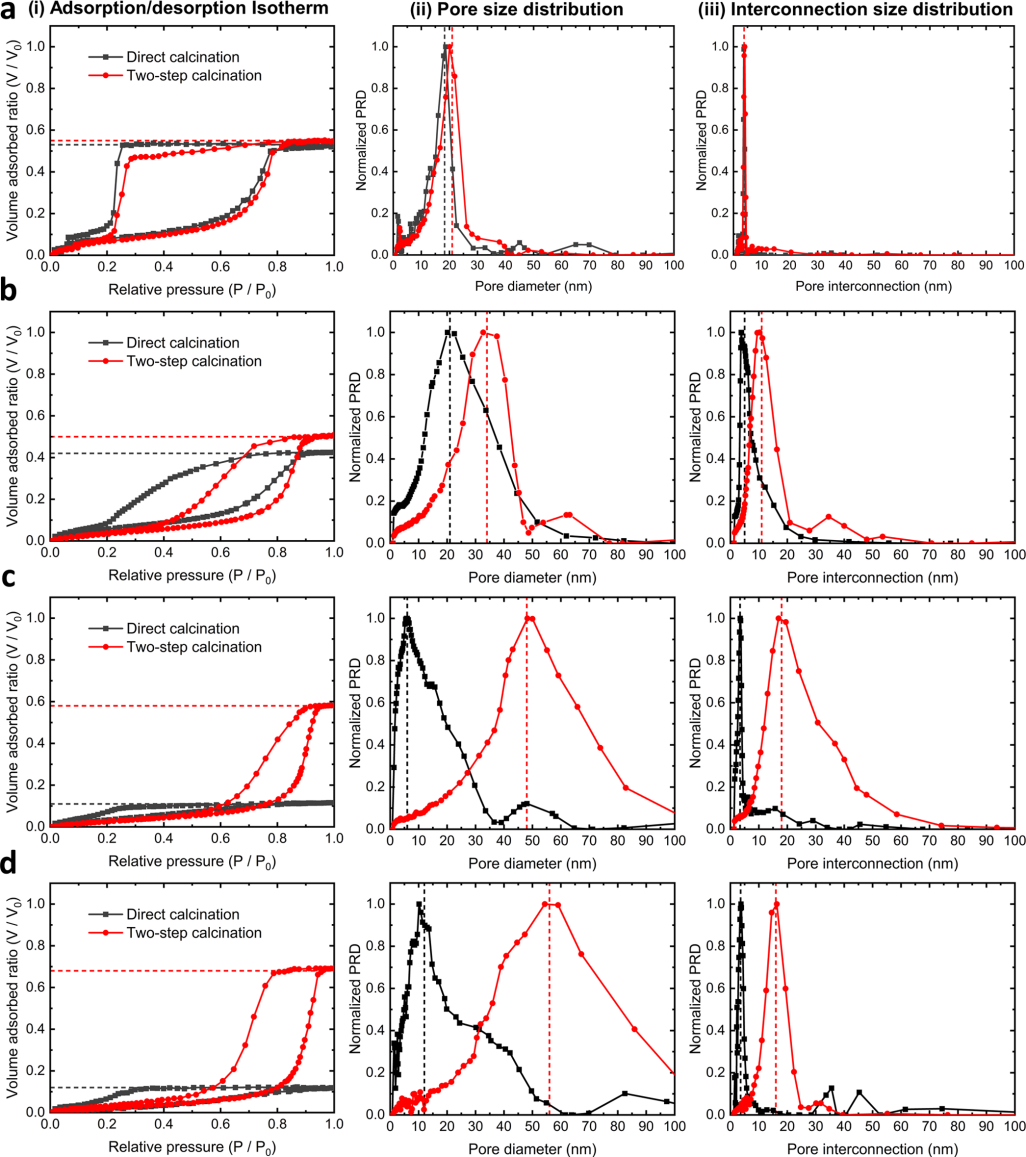
Typical ellipsometric porosimetry isotherms of thin films directly calcined in air (black curves) and after two-step calcination (red curves) of (a) BCP_20_, (b) BCP_30_, (c) BCP_40_, and (d) BCP_50_ films alongside their adsorption/desorption isotherms (i), pore size distribution (ii), and pore interconnection size distribution (iii), respectively. Red and black dashed lines indicate the corresponding film porosity, mean pore size, and mean pore interconnection size.

We next investigated the effect of two-step calcination on the porosity and pore size of the films by ellipsometric porosimetry (EP) using toluene as the adsorptive, as shown in Figure 4. We classified the shape of the physisorption isotherms (Figure 4a–d) as type IV(a) and the hysteresis loop as H2b, according to the IUPAC categories.^43^ This classification is typical of mesoporous materials with ellipsoidal pores interconnected by narrow necks.^22^ The isotherm shape of the two-step calcined films was found to follow similar trends to those directly calcined in air for films BCP_20_ and BCP_30_, indicating that the proposed two-step fabrication process had less impact on the nature of the porous structure when fabricated with low organic content. However, BCP_40_ and BCP_50_ air-calcined films exhibited an H4 hysteresis loop, typical of micro-mesoporous materials,^43^ which we attribute to film collapse during calcination. Porosity values of the films were obtained from the maximum toluene volume adsorbed on each isotherm. For films calcined directly in air, the porosity ranged from 54 ± 2 to 5 ± 4% (Figure 4a–d(i) black curves) for the films BCP_20_ to BCP_50_. In contrast to the expected behaviour, higher content of BCP did not lead to larger porosities, providing evidence for the lack of structural support. Notably, the porosity was consistently higher for samples calcined in two steps, with a range of 56 ± 2 to 70 ± 3% (Figure 4a–d(i), red curves) for equivalent BCP content. Furthermore, argon-annealed films fabricated with 50% block copolymer led to porosities similar to the packing factor of a perfect hexagonal close-packed or a face-centered cubic structure (74%).

The pore size distribution was calculated from the adsorption branch of the isotherms using the modified Kelvin equation,^44^ as depicted in Figure 4a–d(ii). The mean pore size (*D*_ads_) of the two-step calcined films was consistently larger (20 ± 1 to 48 ± 7 nm) compared to films directly calcined in air (17 ± 1 to 8 ± 2 nm) with equivalent organic content (BCP_20_ to BCP_50_). The variation of the pore diameter with polymer content is indicative of dynamic equilibration as seen in bulk behavior.^19^ To assess pore dispersity, information entropy is an unbiased method for comparison between size distributions.^45^ Hence, the uniformity of the pores markedly improved as demonstrated by the smaller values of the normalized information entropy, e.g., 1.25 (BCP_20_) and 1.53 (BCP_40_) for the two-step calcined films in comparison to 1.26 (BCP_20_) and 2.35 (BCP_40_) for direct calcination.

Figure 4a–d(iii) shows the pore interconnection size distribution calculated from the desorption branch of the isotherms. The mean size of the interconnections (*D*_des_) of films treated with the two-step calcination process increased with the organic content up to 21 ± 4 nm (BCP_40_). The ratio between the dimensions of the pore interconnections and pore size also increased at higher organic content, with 0.19 for BCP_20_, 0.29 for BCP_30_, 0.41 for BCP_40_, and 0.31 for BCP_50_. In direct comparison, the ratio was 0.21 for BCP_20_ and 0.18 for BCP_30_ after direct air calcination, while higher organic content led to a collapse of the structure. These findings are particularly valuable for applications requiring mass transfer through the mesoporous network.

**Figure 5:**
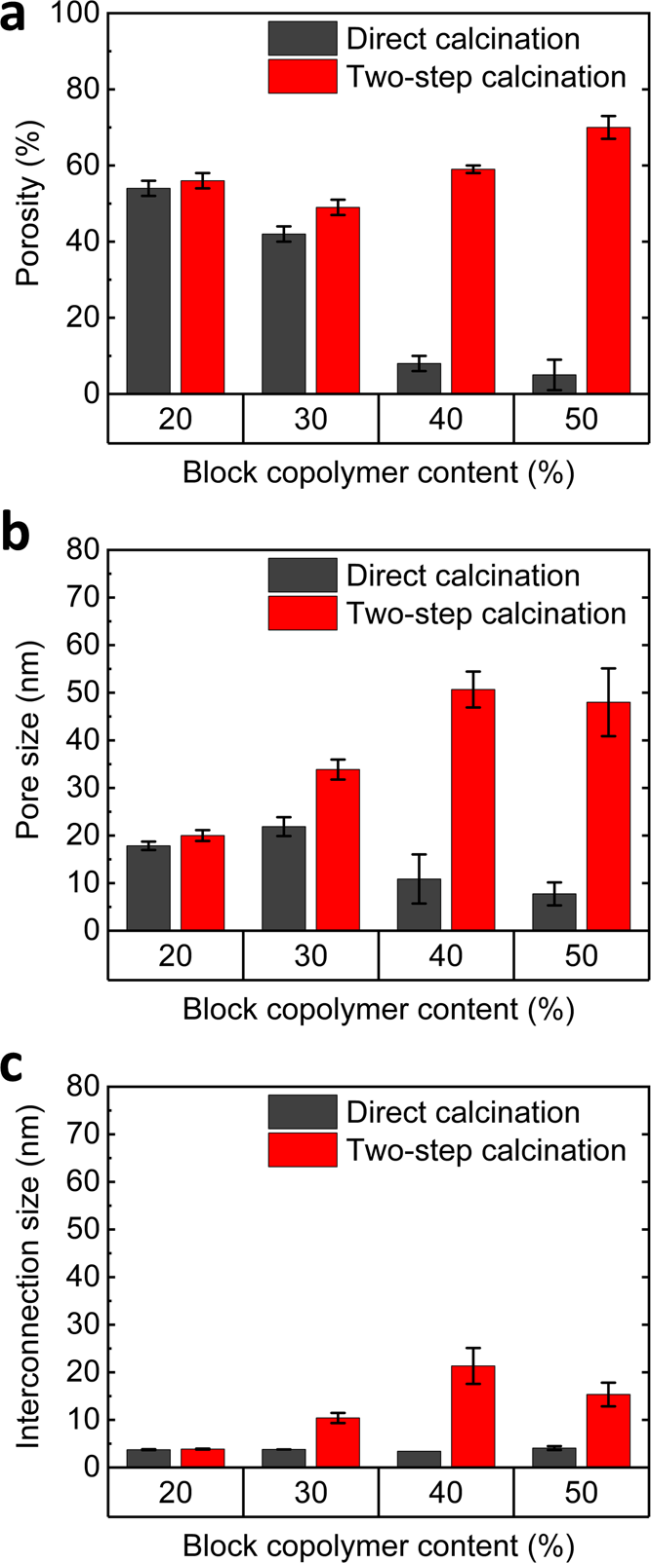
Graphical summary of (a) porosity, (b) pore size, and (c) pore interconnection size obtained after air calcination (black bars) and two-step calcination (red bars) of thin films fabricated with different block copolymer contents.

In summary, thin films initially treated at an elevated temperature in argon atmosphere consistently displayed an increased accessible porosity and lower shrinkage as well as bigger pore size compared to films directly calcined in air. The benefits of two-step calcination on the mesoporous structure were consistent with the BCP content used in the fabrication of thin films. For instance, while BCP_50_ resulted in films with negligible accessible porosity after direct calcination in air, the porosity and pore size were highest for BCP_50_ films calcined in two steps. We attributed this difference to the carbonized portion acting as a scaffold of the inorganic during the condensation at high temperature, thus preventing the collapse of the mesoporous structure due to premature BCP degradation. These findings demonstrate the robustness of the method to preserve the arrangement obtained at the hybrid stage, allowing to fabricate inorganic mesoporous films within a wider range of organic/inorganic ratios than for direct air calcination. Figure 5 and Table 1 summarize all of the structural parameters obtained by SE and EP for three samples of each composition. We also demonstrated the applicability of this fabrication method to mesoporous films fabricated using a BCP with smaller Mn, poly(isobutylene)-*b*-poly(ethylene oxide) PIB-*b*-PEO. In this block copolymer, the hydrophobic PIB block exhibits a lower number of thermally stable sp^2^-hybridized carbon in comparison to PI blocks. We obtained comparable EP results, as shown in Figures S2 and S3.

**Table 1:** Thin Film Structural Parameters Obtained from Spectroscopic Ellipsometry and Porosimetry Measurements of Three Samples of Each Composition^1^

sample	calcination process	thickness before calcination [nm]	thickness after calcination [nm]	final thickness [% initial thickness]	uniaxial contraction [%]	porosity [vol %]	mean pore size *D*_ads_ [nm]	normalized information entropy	mean pore interconnections size *D*_des_ [nm]
BCP_20_	two-step	1216 ± 34	439 ± 10	36 ± 0.8	64 ± 0.8	56 ± 2	20 ± 1	1.25	3.8 ± 0.1
BCP_20_	direct	1207 ± 25	435 ± 5	36 ± 1.1	64 ± 1.1	54 ± 2	17 ± 1	1.26	3.7 ± 0.1
BCP_30_	two-step	785 ± 5	268 ±0	34 ± 0.8	66 ± 0.8	49 ± 2	34 ± 2	1.42	10.0 ± 1
BCP_30_	direct	799 ± 4	217 ± 6	27 ± 0.7	73 ± 0.7	42 ± 2	22 ± 2	1.96	4 ± 0.1
BCP_40_	two-step	639 ± 3	214 ± 2	34 ± 0.4	66 ± 0.4	59 ± 1	51 ± 4	1.53	21 ± 4
BCP_40_	direct	640 ± 6	102 ± 3	16 ± 0.5	84 ± 0.5	8 ± 2	11 ± 5	2.35	3 ± 0.1
BCP_50_	two-step	535 ± 6	151 ± 5	28 ± 1.0	72 ± 1.0	70 ± 3	48 ± 7	1.48	15 ± 2
BCP_50_	direct	530 ± 1	72 ± 5	14 ± 0.9	86 ± 0.9	5 ± 4	8 ± 2	1.98	4 ± 0.4

1 Error corresponds to the standard deviation of three samples.

### Effect of Two-Step Calcination in the Mesoporous Order

**Figure 6:**
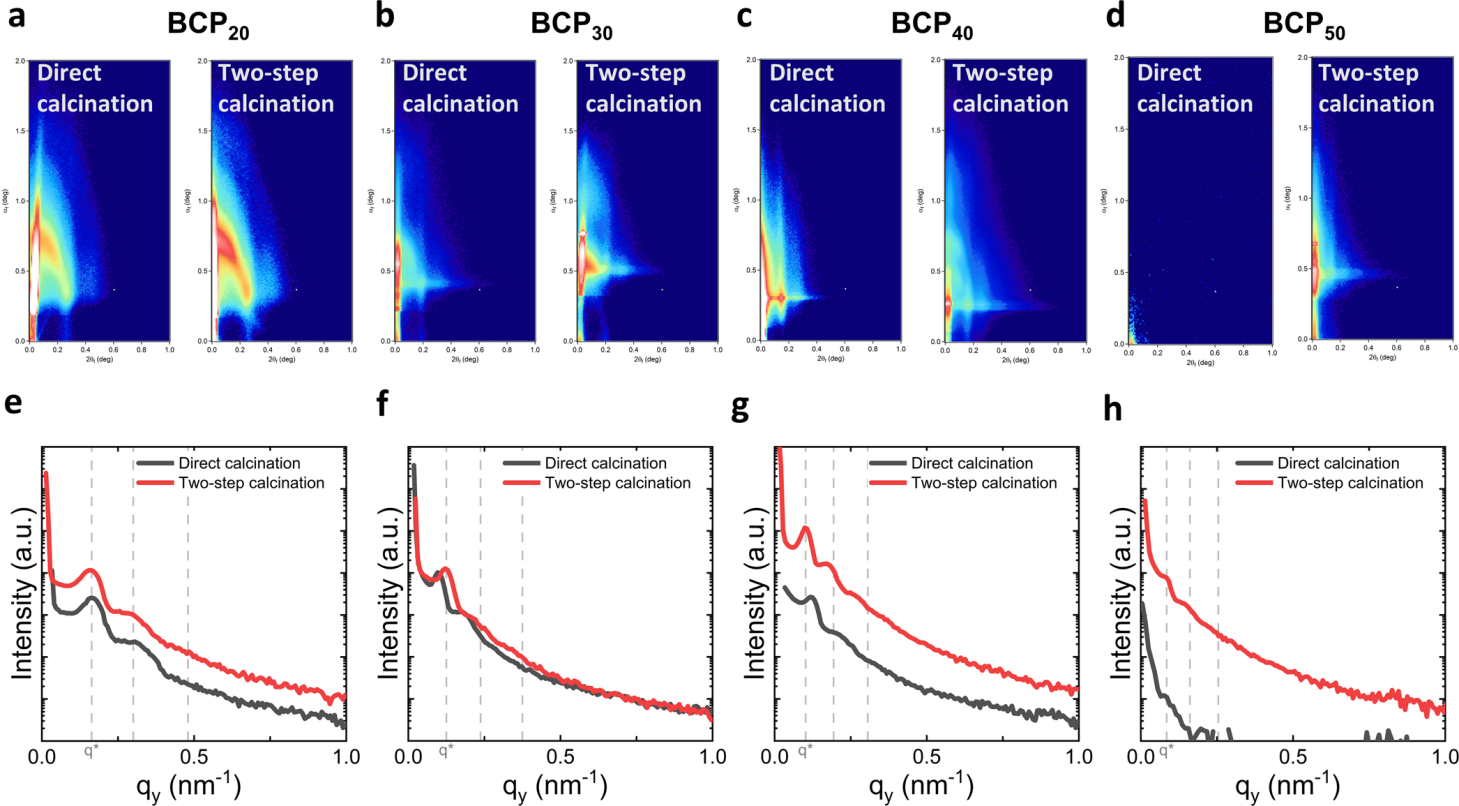
Thin film characterization by GISAXS of the mesoporous films (a, e) BCP_20_, (b, f) BCP_30_, (c, g) BCP_40_, and (d, h) BCP_50_. (a–d) 2D GISAXS scattering patterns and (e–h) in-plane GISAXS line cuts, *q_y_*, of samples directly calcined in air and after two-step calcination. Dashed lines correspond to nominal peak position ratios (*q*/q*) of 1, 1.9, and 3.

Grazing-incidence small-angle X-ray scattering (GISAXS) is a nondestructive characterization technique that allows obtaining structural information from mesoporous thin films, such as the packing structure, center-to-center distance (*D*_C–C_), and order domains.^46^ Since the footprint of the X-ray beam at grazing incidence is typically of several mm^2^, GISAXS provides structural information over a relatively large sample area, compared to atomic force or scanning electron microscopy. Figure 6a–d shows the scattering patterns for films calcined only in air and via the two-step protocol, respectively. The integration of the GISAXS line cutting along the in-plane direction (*q_y_*) provides information of the horizontal mesopore arrangement (Figure 6e–h). We consistently found that all two-step thin films exhibited in-plane order, with two to three intensity peaks with the approximate angular ratios of 1, 1.9, and 3, which are consistent with a number of different symmetry groups, including body-centered cubic (BCC), a face-centered cubic (FCC) with stacking fault,^47^ hexagonal close-packed (HCP) mesoporous order,^48^ and local paracrystalline ordering from randomly packed spheres.^49^ This was in contrast to the absence of the diffraction peak obtained for the sample BCP_50_ directly calcined in air, indicating the loss of mesoporous order. Sample BCP_40_ directly calcined displayed predominant in-plane ordering with a larger *q*-spacing in the out-of-plane direction, consistent with uniaxial contraction as observed elsewhere with mesoporous titania films,^50,51^ and other materials.^52,53^ Such sample contraction is expected to reduce the extent of ordering obtained in the hybrid state. To this end, two-step calcination is expected to retain an improved overall ordering. Consistently, the GISAXS scattering pattern of a mesoporous film BCP_50_ in the hybrid state and after two-step calcination (Figure S4) showed that the in-plane periodicity was retained after two-step calcination, as denoted by the peaks *q_y_* in the same position before and after calcination. The lack of in-plane displacement suggests that the thin films were well-adhered to the substrate and predominantly contracted uniaxially (out-of-plane shrinkage) during calcination. Finally, we found that the in-plane grain size, i.e., the Scherrer domain size^54^ calculated from the width of the first peak (q*) normalized by the in-plane *D*_C–C_, was generally larger for the two-step calcined samples compared with their air-calcined counterparts, suggesting a better retention of ordering. We obtained similar trends on mesoporous films fabricated with the BCP PIB-*b*-PEO, as shown in Figure S5.

Percolation paths through a porous structure are important for diffusion-related applications.^10^ It is reasonable to assume that the higher degree of order obtained by two-step calcination of samples fabricated with high organic content leads to improved accessibility of the porous network; we relate this to less distorted percolation paths consistent with the degree of interconnectedness determined from physisorption. Table 2 summarizes the in-plane center-to-center distance (*D*_C–C_), Scherrer domain size, and extent of order calculated from the GISAXS patterns.

**Table 2:** Thin Film Structural Parameters Obtained by the Analysis of the GISAXS Patterns

sample	calcination process	first Bragg peak q* [nm^–1^]	in-plane *D*_C–C_ [nm]	Scherrer domain size *D*_Sch_ [nm]	extent of order [pores]
BCP_20_	two-step	0.163	38	87	2.3
BCP_20_	direct	0.167	38	116	3.1
BCP_30_	two-step	0.113	55	423	7.6
BCP_30_	direct	0.0995	63	197	3.1
BCP_40_	two-step	0.102	61	210	3.4
BCP_40_	direct	0.123	51	167	3.3
BCP_50_	two-step	0.0848	74	339	4.6
BCP_50_	direct				

**Figure 7:**
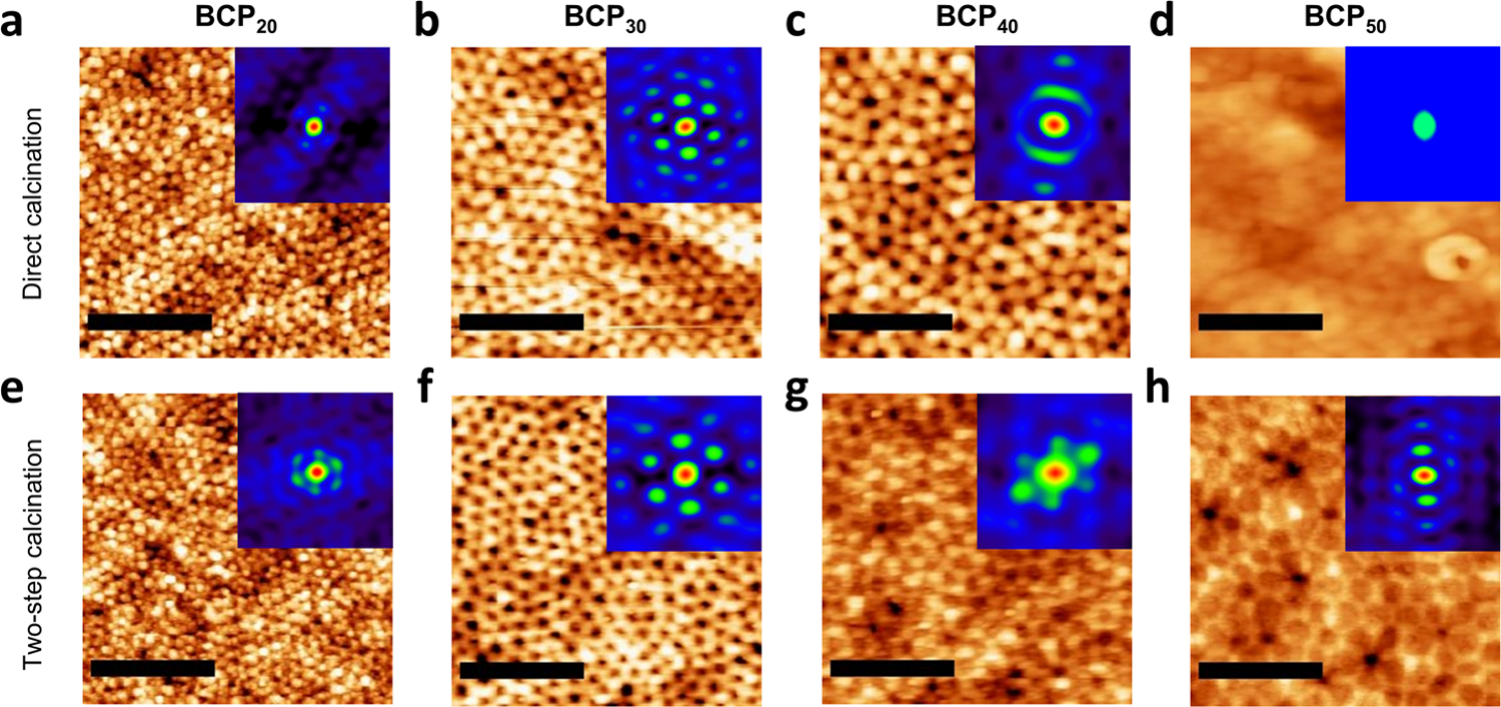
AFM images of the mesoporous films (a–d) after direct calcination in air and (e–h) after the two-step calcination process. The insets correspond to the 2D spatial distribution function to evaluate pore ordering. Scale bar: 400 nm.

AFM micrographs of the thin films’ top surface confirmed the structural pore arrangement, in-plane *D*_C–C_, and pore size obtained by EP and GISAXS, as shown in Figure 7. To evaluate the mesoporous arrangement of the samples, AFM micrographs were analyzed to obtain the 2D spatial distribution function (SDF), an alternative measure for 2D structural order with distinct advantages over the standard 2D fast Fourier transform (FFT) in case of limited periodicity (see Figure S6 for the corresponding 2D FFT). The higher number of concentric hexagonal rings displayed in the 2D SDF (Figure 7 insets) of the two-step calcined samples suggests that the pores on the film surface exhibit a higher degree of order in comparison to samples calcined directly in air, which is in line with previous GISAXS observations. A similar degree of order was observed in SEM images of mesoporous thin films fabricated with PIB-*b*-PEO (Figure S7).

Higher-magnification SEM micrographs (Figure S8) revealed the interconnected nature of the mesoporous structure obtained by the coassembly of aluminosilicates and block copolymers. We also noticed that the surface of the mesoporous films fabricated by either direct or two-step calcination was homogeneous and crack-free for the film thicknesses studied herein (<500 nm after calcination). Condensation of the sol–gel occurs simultaneously in all directions of the film structure. Having a film firmly attached to the substrate generates in-plane stresses that are normally released by the mesopores, which act as a relaxing agent.^55^ Failure to release the in-plane tensile stress results in the formation of cracks on the macroscale, which is usually observed for films of micrometer thickness.^35^ The fact that we did not observe the formation of cracks suggests that the in-plane stresses were effectively released by deformation of the pores in the in-plane direction, avoiding the formation of cracks.^55^ In consequence, the pore shape is expected to be ellipsoidal rather than a perfect spherical shape. Figure S9 shows the cross section of a mesoporous film illustrating this effect. We refer to our previous work on structural characterization of mesoporous films for a guidance on the determination of in-plane and out-of-plane pore dimensions from ellipsometric porosimetry.^22^

We note that during this study, similar results were obtained using nitrogen as non-oxidizing atmosphere during formation of the carbon scaffold. However, in the case of nitrogen, the formation of nitrides at high temperatures under certain experimental conditions must be considered.

### Enzyme Storage into Mesoporous Thin Films

**Figure 8:**
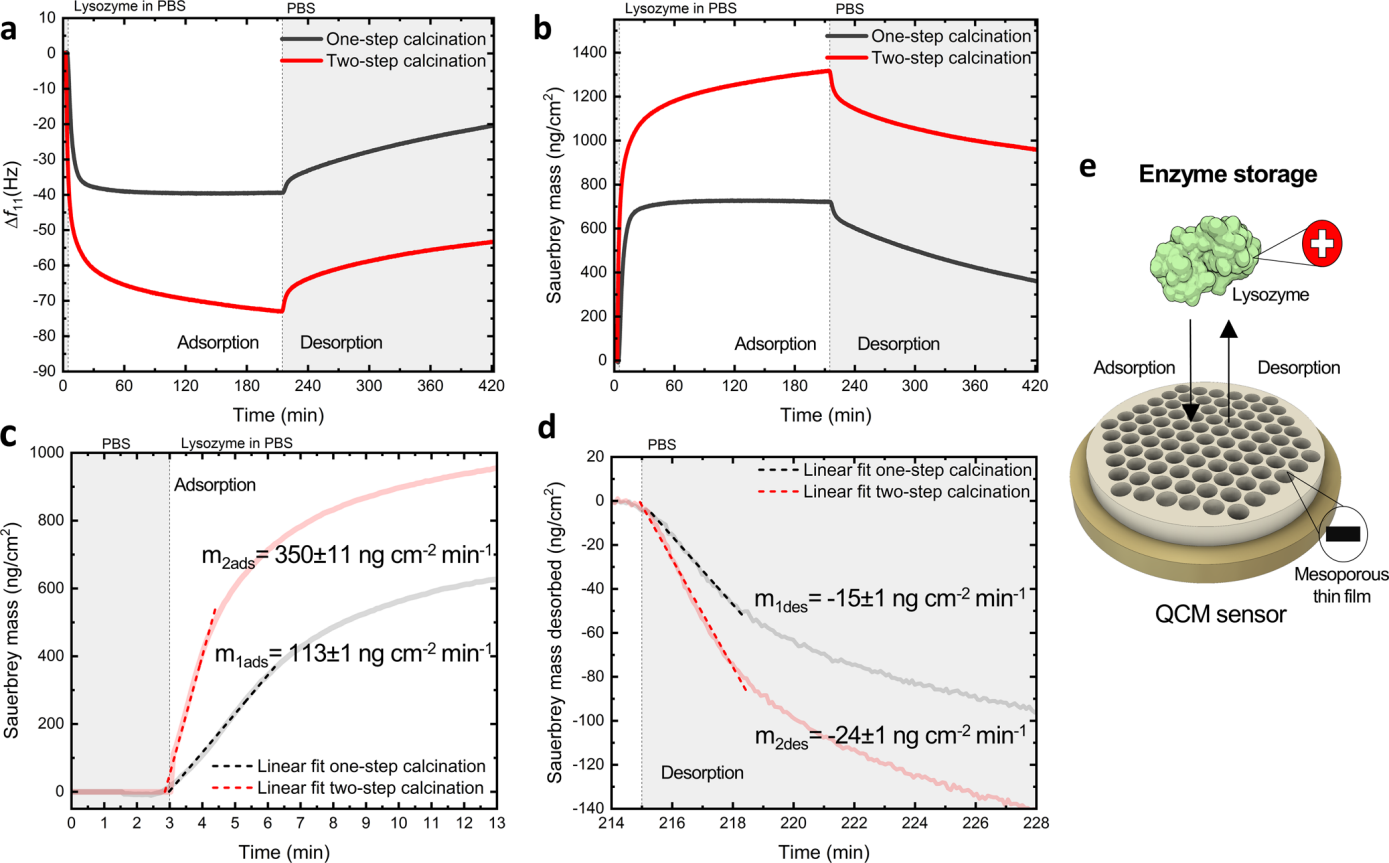
Real-time adsorption/desorption of lysozyme into mesoporous thin films. (a) Comparative frequency responses (11th harmonic) and (b) mass changes of one-step and two-step calcined films toward 2 mg mL^–1^ of lysozyme in PBS buffer. Zoom into the first minutes of protein (c) adsorption and (d) desorption, displaying the rate (slope of the linear fit) to compare physisorption dynamics. (e) Schematic of the enzyme storage into mesoporous thin films measured by QCM.

To study the effect of the mesoporous structure obtained by two-step calcination in the mass transport of large molecules, we measured in real time the enzyme physisorption into the mesoporous films using a quartz crystal microbalance (QCM). Because the aluminosilicate mesoporous films are negatively charged at neutral pH (see Figure S10), lysozyme, a globular protein with enzymatic and antimicrobial properties,^56^ was chosen as a model system. This protein is positively charged at pH 7.3 (isoelectric point pI = 11)^57^ and exhibits suitable dimensions (3.0 × 3.0 × 4.5 nm^3^)^58^ for the herein studied mesoporous architecture. We used BCP_40_ mesoporous films for enzyme storage because it displayed the most favorable ratio between the dimensions of the pore interconnections and pore size. To this end, we prepared mesoporous films onto silica-coated QCM sensors and measured the frequency changes in real time when exposed to 2 mg mL^–1^ of lysozyme in PBS buffer, as shown in Figure 8a. We then related the frequency changes to the enzyme mass using the Sauerbrey equation,^59^ as illustrated in Figure 8b.

We found that two-step calcined mesoporous films adsorbed more lysozyme (1320 ng cm^–2^) than the one-step calcined films (720 ng cm^–2^) after 3.5 h of exposure to the enzyme-rich solution. The adsorbed mass difference (∼45%) was in proportion to the porosity difference of the films (see Figure S11). This observation is in good agreement with previous studies showing that protein loading efficiency increases with pore volume in mesoporous silica materials.^60,61^Figure 8c shows the first minutes of enzyme adsorption into the films. The three times faster adsorption kinetics observed in two-step calcined films (adsorption rate *m*_2ads_ = 350 ± 11 ng cm^–2^ min^–1^) with respect to one-step calcined films (adsorption rate *m*_1ads_ = 113 ± 1 ng cm^–2^ min^–1^) corroborate the improved pore connectivity and pore access obtained with the two-step fabrication protocol. Similarly, two-step calcined films desorbed lysozyme nearly two times quicker (desorption rate *m*_2des_ = −24 ± 1 ng cm^–2^ min^–1^) than directly calcined films (desorption rate *m*_2des_ = −15 ± 1 ng cm^–2^ min^–1^) when exposed to PBS buffer, as shown in Figure 8d. Please note that remnant carbon species that are not removed by air calcination could introduce negative surface charges,^23,62^ which may favor adsorption kinetics of positively charged molecules, with the opposite effect on desorption. The improved adsorption and desorption kinetics observed in two-step calcined films suggest that the effect of residual carbon, if present, is negligible in the adsorption/desorption of large molecules.

These findings provide evidence that the improved structural order with respect to pore shape and pore interconnectivity enhances accessibility to the porous network of large molecules. The less restricted porous structures obtained in two-step calcined films allow to minimize the attractive electrostatic interactions between lysozyme and the aluminosilicate surface, easing protein desorption. The ability to store and release large molecules from the mesoporous structure is important for various use cases such as in antibacterial coatings,^63^ glucose sensors,^64^ and biocatalysts.^65^ In consequence, processing mesoporous films at a high temperature in argon prior to air calcination offers a clear benefit for these applications. Figure 8e schematizes the enzyme storage application using mesoporous-coated QCM sensors. Finally, we want to emphasize that our findings are equally applicable to related approaches of material templating, such as other BCP systems with sp^2^-hybridized carbon building blocks and colloidal coassembly,^66^ in addition to sol–gel precursors with similar reaction kinetics (e.g., siloxanes). Further work is needed to establish the suitability of transient carbon scaffolds to enable high-temperature crystallization under confinement in thin films, similar to previous observations in bulk materials.^38^

## Conclusions

We have demonstrated that a two-step calcination protocol, composed of high-temperature treatment in argon, followed by air calcination, reduces the uniaxial contraction of inorganic mesoporous thin films fabricated by supramolecular coassembly in comparison with the common protocol involving air calcination immediately after film deposition.

The two-step calcined mesoporous films retain higher porosity, display a larger and more uniform pore size, and exhibit an improved porous order than one-step calcined films, as demonstrated here for a wide range of organic/inorganic ratios and two block copolymers (PI-*b*-PEO, PIB-*b*-PEO). While uniaxial shrinkage occurs in direct air calcination protocols due to the concurrent sol–gel condensation and premature degradation of the organic BCP host, the herein proposed fabrication method allows to retain the structural support of the carbonized organic host during the sol–gel condensation. This effect is particularly pronounced in films containing a larger volume fraction of block copolymer, enabling to achieve porosities near 70% at compositions where the removal of the organic structure-directing agent would otherwise lead to the collapse of the inorganic network. Thus, two-step calcination enables access to a broader library of pore characteristics with the same material system using different organic–inorganic ratios.

Furthermore, larger ratios between the dimensions of the pore interconnections and the pore sizes can be achieved. In consequence, the mesoporous architectures obtained by two-step calcination display enhanced mass-transport properties of large molecules demonstrated here for lysozyme adsorption as an example of enzyme storage but offering favorable performance in a broad range of applications.

## Experimental Section

### Reagents

Poly(1,4-isoprene)-*b*-poly(ethylene oxide) block copolymer (polydispersity: 1.01, *M*_n_ PI(48)-*b*-PEO(12) kg mol^–1^) was purchased from Polymer Source Inc. PIB_39_-*b*-PEO_36_ block copolymer (polydispersity 1.26, *M*_n_ 4.85 kg mol^–1^) was provided by BASF. Toluene (99.9%), 1-butanol (99.4%), aluminum tri-sec-butoxide (97%), (3-glycidyloxypropyl)-trimethoxysilane (GLYMO) (≥98%), potassium chloride (KCl) (≥99.9%), hexaammine ruthenium(III) chloride ([Ru(NH_3_)_6_]Cl_3_) (98%), and lysozyme from chicken egg white (lyophilized powder, protein ≥ 90%, ≥40 000 units mg^–1^ protein) were purchased from Sigma-Aldrich. Potassium ferricyanide (K_3_[Fe(CN)_6_]) (>99%) was purchased from ACROS Organics. Potassium ferrocyanide (K_4_[Fe(CN)_6_]) (>98.5%) was purchased from Honeywell. Phosphate buffered saline (PBS) in tablets was purchased from OXOID. All chemicals were used without further purification.

### Preparation of Aluminosilicate Sol–Gel Stock Solution

The aluminosilicate stock solution was prepared by mixing and stirring in an ice bath 0.32 g of aluminum tri-sec-butoxide, 2.8 g of GLYMO, and 20 mg of KCl. After 15 min of stirring, 0.135 mL of a 10 mM HCl solution was added dropwise to start the hydrolysis of the precursors and left for another 15 min in the ice bath. The mixture was then removed from the ice bath and stirred at room temperature for 15 min. HCl (0.85 mL, 10 mM) was added to the solution and stirred for 20 min to complete the hydrolysis. The final solution was filtered with a 0.2 μm cellulose syringe filter and dissolved with 2.135 mL of toluene/1-butanol (72.84/27.16 wt %) to obtain a concentration of 1 g mL^–1^ of aluminosilicate. The mixture was then kept refrigerated at 5 °C.

### Preparation of the Block Copolymer Stock Solution

PI-*b*-PEO was dissolved in an azeotropic solution of toluene/1-butanol (72.84/27.16 wt %) at a concentration of 40 mg mL^–1^ and used without further filtration. PIB-*b*-PEO received from BASF was dissolved in toluene/1-butanol at a concentration of 32 mg mL^–1^ and subsequently filtered with a 0.2 μm cellulose syringe filter.

### Fabrication of Mesoporous Aluminosilicate Thin Films by Block Copolymer Coassembly

First, the BCP stock solution was mixed with the aluminosilicate sol–gel stock solution in volumes described in Table 3, producing the so-called hybrid solution and left mixing in a shaker for 30 min prior to use. The hybrid solution (30 μL) was spin-coated (2000 rpm, 20 s, Laurell WS 650 MZ) onto silicon, silica-coated QCM sensors (5 MHz 14 mm Cr/Au/SiO_2_, Quartz PRO), FTO-coated glass (TEC 6, Pilkington), or Au-coated silicon substrates to produce the thin films. All substrates were plasma-treated in oxygen before deposition (300 s, 100 W, 0.33 mbar, Diener Electronic “Pico”) to activate the surface and remove organic contaminants. Thin films were subsequently calcined. “One-step calcination” films were calcined in air at 450 °C (30 min, 5 °C min^–1^). “Two-step calcination” films were first annealed in argon at 450 °C (30 min, 5 °C min^–1^) in a tubular furnace. Samples were let to cool inside the furnace. Films were subsequently air-calcined at 450 °C (30 min, 5 °C min^–1^) in a muffle furnace and let to cool inside the furnace.

**Table 3:** Block Copolymer and Aluminosilicate Volumes Used to Generate Hybrid Solutions of various BCP weight fractions

sample	block copolymer	block copolymer content [wt %]	BCP stock solution [μL]	aluminosilicate stock solution [μL]
BCP_20_	PI*-b*-PEO	20	375	120
BCP_30_	PI*-b*-PEO	30	375	70
BCP_40_	PI*-b*-PEO	40	375	45
BCP_50_	PI*-b*-PEO	50	375	30
BCP_20*_	PIB*-b*-PEO	20	469	120
BCP_30*_	PIB*-b*-PEO	30	469	70
BCP_40*_	PIB*-b*-PEO	40	469	45

### Fourier Transform Infrared Spectroscopy

FTIR spectra were measured in reflection mode on thin films fabricated onto Au-coated silicon substrates using an AIM-9000 infrared microscope coupled with an IRTracer-1000 FTIR spectrophotometer (Shimadzu). Atmospheric and baseline corrections were performed with the software Lab Solutions IR (Shimadzu). Annealing was performed in samples placed on top of a hot plate by heating from room temperature to 450 °C with a heating ramp of 5 °C min^–1^ to simulate the conditions of direct calcination. Once a temperature of 150 °C was reached, thin films were sequentially removed from the hot plate every 30 °C of increment, 2 min after the target temperature was reached.

### Raman Spectroscopy

Raman spectroscopy study was carried out using a Renishaw 1000 spectrometer equipped with a 633 nm laser (1.9 eV, 1.0 mW) and coupled to a microscope with a 50× lens. The Raman system was calibrated using a silicon reference.

### Spectroscopic Ellipsometry (SE), Ellipsometric Porosimetry (EP), and Environmental Ellipsometric Porosimetry (EEP)

SE, EP, and EEP were applied to the characterization of mesoporous films as previously reported.^67^ In short, samples were processed on silicon substrates and studied on a Semilab SE2000 ellipsometer with variable angle and a spectral range between 300 and 989 nm. SE for film thickness measurements were carried out at an incident angle of 73°. *In situ* spectroscopic ellipsometry was measured at an incident angle of 65° in a customized chamber that allowed atmospheric and thermal control up to 300 °C,^68^ deploying a heating ramp of 5 °C min^–1^ for one-step and two-step calcined films. A flow controller (model F-201CV, Bronkhorst) was used to flow argon into the chamber (0.1 L min^–1^). The integrated SEA software (Semilab) served to fit the experimental data, Ψ and Δ. Film thickness and refractive index were obtained using a Cauchy dispersion law and Levenberg–Marquardt algorithm (LMA), with a fit quality *R*^2^ > 0.95 for all of the measurements. Isotherms were measured in an enclosed chamber with automatic vacuum and solvent atmosphere control (Semilab). Spectroscopic ellipsometry was automatically measured while increasing (adsorption) and decreasing (desorption) the solvent partial pressure in the chamber. Adsorption and desorption isotherms were derived from the changes in refractive index upon toluene and water adsorption and desorption, respectively. The refractive index was modeled using the Lorentz–Lorentz effective medium approximation using a simplex fitting algorithm with a tolerance of 1 × 10^–6^ and a maximum of 1000 iterations. Pore size distribution and pore interconnection size distribution were obtained using the modified Kelvin equation in the adsorption and desorption isotherms, respectively. The contact angle between aluminosilicate–toluene and aluminosilicate–water was assumed to be zero (perfect wetting). Normalized information entropy of the pore size distribution was calculated using a method described elsewhere,^45^ using a bin width of 0.1.

### Grazing-Incidence Small-Angle X-ray Scattering

GISAXS measurements were performed on a SAXSLab Ganesha instrument. A Xenocs GeniX3D microfocus source was used with a Cu target to produce a monochromatic beam with a wavelength of 0.154 nm. The instrument was calibrated before use with a National Institute of Standards and Technology (NIST) Si reference material 640d with a reference peak position of 2θ = 28.44°, where 2θ represents the total scattering angle. A Pilatus 300K detector (Dectris) collected the two-dimensional (2D) scattering pattern. The detector exhibits a nominal pixel dimension of 172 × 172 μm^2^. The SAXS data were acquired with an X-ray flux of ∼4.1 million photons per second with a sample-to-detector distance of 1052 mm. Experiments were conducted with samples rotated to an incident angle of 0.2° with respect to the incident beam. All samples were measured for 1 h. GISAXS data analysis was performed using FitGISAXS^69^ software.

### Scanning Electron Microscopy (SEM)

SEM images provided in the Supporting Information were taken in an Xbeam 540 FIB/SEM (ZEISS) directly on aluminosilicate mesoporous films without any metallic coating. Images were captured using an acceleration voltage between 0.5 and 2 kV and a working distance between 0.9 and 1 mm. The 2D spatial distribution function was calculated with the software CORDERLY.^70^

### Atomic Force Microscopy (AFM)

AFM images were obtained in tapping mode with a Bruker Dimension Icon AFM instrument with a Bruker ScanAsyst Air Probe with a nominal tip radius of 2 nm.

### Electrochemical Measurements

All measurements were obtained with a potentiostat (Reference 600+, Gamry) in a three-electrode setup. CV measurement conditions: potential range [Ru(NH_3_)_6_]^2/3+^: 0 to −0.4 to 0.2 V; potential range [Fe(CN)_6_]^3/4–^: 0 to −0.6 to 0.6 V; scan rate: 100 mV s^–1^. EIS measurement conditions: frequency range: 0.1 Hz to 100 kHz, amplitude 5 mV, DC voltage 0 V vs OC. The reference, counter, and working electrode were Ag/AgCl, a platinum wire (0.4 mm diameter), and FTO-coated glass containing the mesoporous film (area: 0.5 cm^2^), respectively. The negatively charged electrolyte [Fe(CN)_6_]^3/4–^ was a mixture of 2 mM potassium ferrocyanide and 2 mM potassium ferricyanide in 0.1 M PBS. The positively charged electrolyte [Ru(NH_3_)_6_]^2/3+^ was 1 mM hexaammineruthenium(III) chloride in 0.1 M PBS. Electrochemical measurements were performed on films directly after fabrication without pretreatment and only with the electrolyte and pH mentioned above. Measurements were analyzed and fitted with the software Gamry Echem Analyst.

### Enzyme Storage

Enzyme storage was studied with a quartz crystal microbalance (Q-Sense E4 instrument, Biolin Scientific) on BCP_40_ mesoporous films prepared onto silica-coated QCM sensors (5 MHz 14 mm Cr/Au/SiO_2_, Quartz PRO) with an active area of 0.79 cm^2^. In a continuous measurement, lysozyme adsorption was induced by pumping 2 mg mL^–1^ of lysozyme in 0.1 M PBS buffer (pH 7.3) into the QCM chamber, and subsequent lysozyme desorption was induced by pumping PBS buffer into the chamber. Lysozyme and PBS were pumped into the QCM chamber at a flow rate of 30 μL min^–1^. Frequency analysis, conversion to the Sauerbrey mass using the composite Sauerbrey of the frequency harmonics *f*_3_, *f*_5_, *f*_7_, *f*_9_, *f*_11_, and *f*_13_, and validation of the model were performed with the software QSense Dfind (Biolin Scientific).

## Supporting Information Available

Data related to the block copolymer PIB-*b*-PEO; GISAXS; and cross-sectional film BCP_50_ PI-*b*-PEO; and electrochemical characterization mesoporous aluminosilicate (PDF)
