# Supplementary material for: Enhanced Structural Control of Soft-Templated Mesoporous Inorganic Thin Films by Inert Processing Conditions
Source: ACS Appl Mater Interfaces. 2022 Dec 12;14(50):56143–55. doi: 10.1021/acsami.2c18090 (PMC9782354; doi:10.1021/acsami.2c18090)
Supplement: Supplementary file 1 — am2c18090_si_001.pdf [file am2c18090_si_001.pdf]

SUPPORTING INFORMATION

DOI: 10.1021/acsami.2c18090

Enhanced structural control of soft-templated mesoporous inorganic thin films by inert processing conditions

*Maximiliano Jesus Jara Fornerod[a], Alberto Alvarez-Fernandez[a], Eric Williams [b], Maximilian W.A. Skoda[c], Beatriz Prieto-Simon[d,e], Nicolas H. Voelcker[,f,g], Morgan Stefik [b], Marc-Olivier Coppens[a,h], Stefan Guldin[a]**

[a]Department of Chemical Engineering, University College London, UK.

[b]Department of Chemistry and Biochemistry, University of South Carolina, Columbia, SC, USA

[c]ISIS Pulsed Neutron and Muon Source, Rutherford Appleton Laboratory, Harwell, Oxfordshire, UK.

[d] Department of Electronic Engineering, Universitat Rovira i Virgili, Tarragona, Spain

[e] ICREA, Barcelona, Spain

[f] Monash Institute of Pharmaceutical Sciences, Monash University, Victoria, Australia

[g] Melbourne Centre for Nanofabrication, Victorian Node of the Australian National Fabrication Facility, Clayton, Victoria, Australia

[h]Centre for Nature Inspired Engineering, University College London, UK.

*corresponding author. Email address: s.guldin@ucl.ac.uk

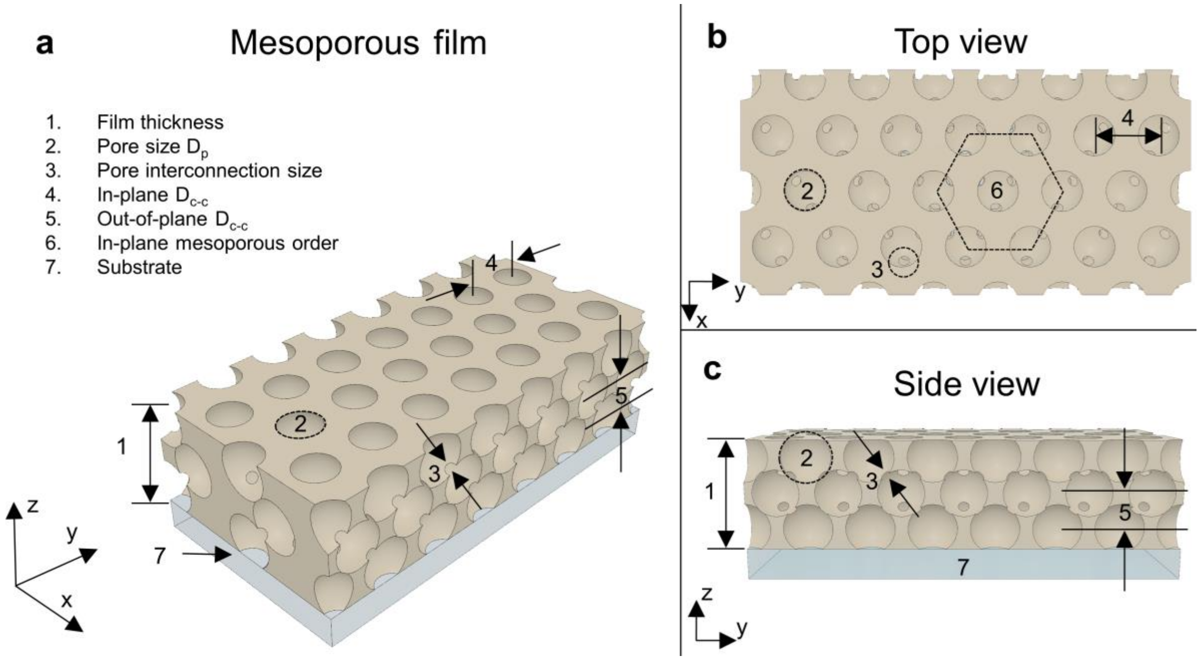

Figure S 1. A) Schematic of a mesoporous film describing the main structural parameters used in in this work. B) Top view and C) side view of the film.

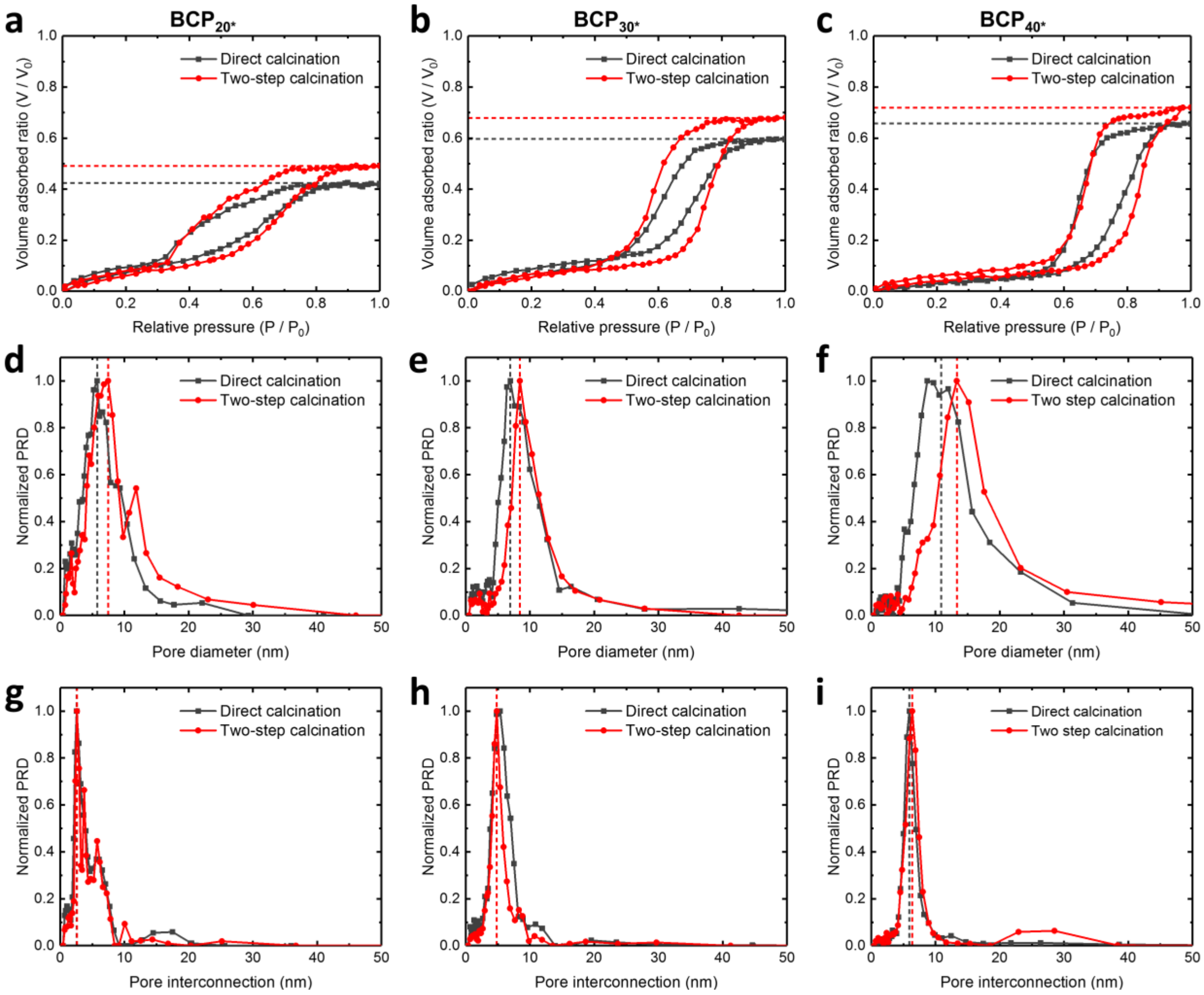

Figure S2. Environmental ellipsometric porosimetry isotherms of thin films directly calcined in air (black curves) and after two-step calcination (red curves) of the films BCP_{20*}, BCP_{30*} and BCP_{40*} fabricated with PIB-*b*-PEO in the hybrid mixture alongside with their pore size distribution (d-f) and pore interconnection size distribution (g-i) respectively. Red and black dashed lines indicate the corresponding film porosity, mean pore size and mean pore interconnection size.

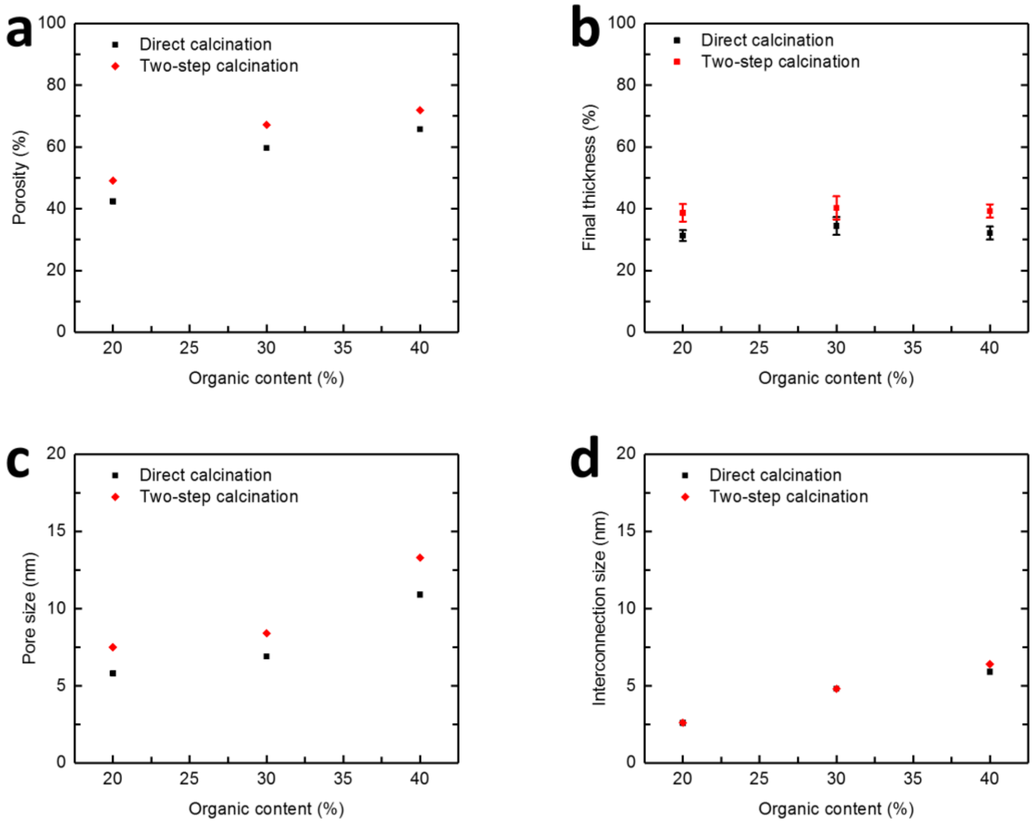

Figure S3. Structural parameters obtained from environmental EP measurements on thin films BCP_{20*}, BCP_{30*} and BCP_{40*} with increasing content of the block copolymer PIB-*b*-PEO: (a) porosity, (b) film thickness, (c) pore size and (d) interconnection size. Thin films were prepared using the two-step calcination process (red dots) and direct air calcination (black dots).

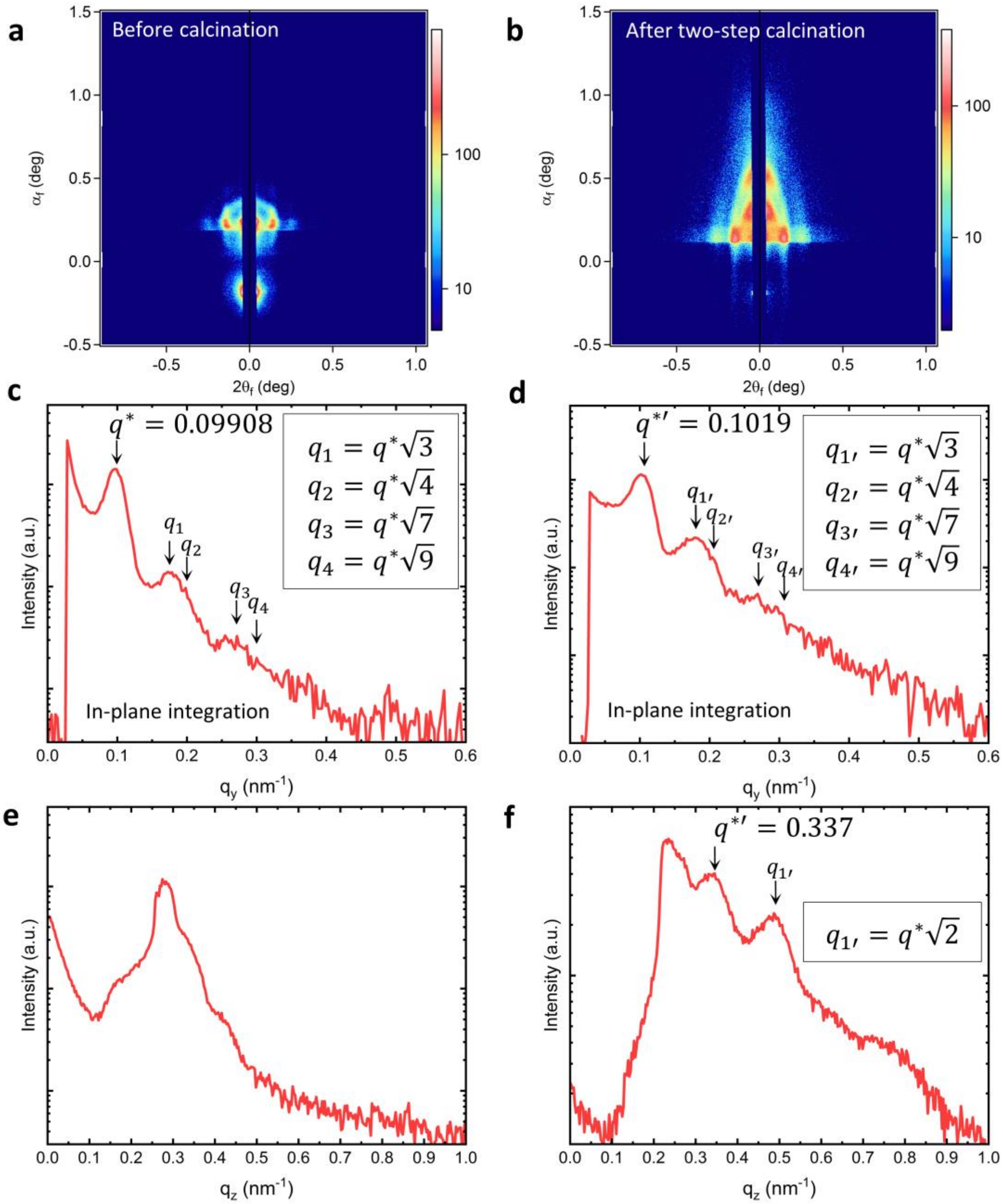

Figure S4. GISAXS scattering patterns of a BCP_{50} mesoporous film (A) before calcination, i.e. hybrid film, and (B) after two-step calcination. In-plane line-cuts integration (q_y) (C) before calcination and (D) after calcination shows the Bragg peaks in the positions of a hexagonal close-packed pore arrangement. Out-of-plane line-cuts integration (q_z) (E) before calcination and (F) after calcination.

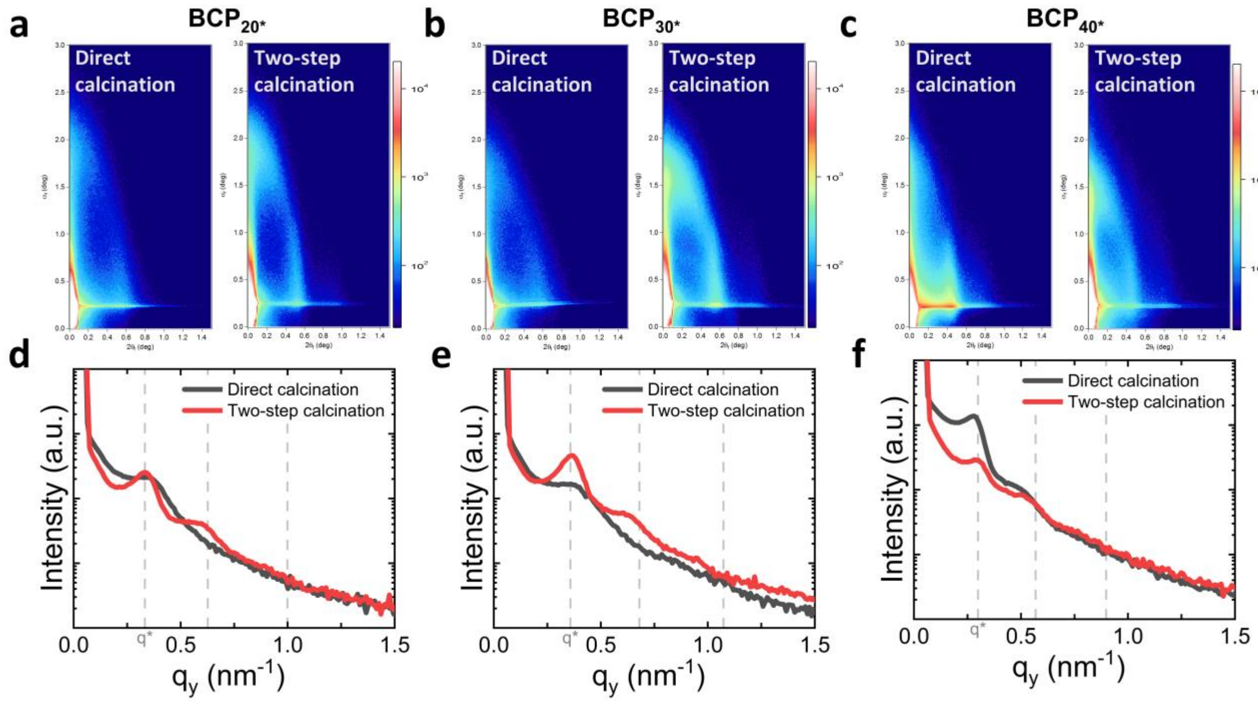

Figure S5. Grazing-Incidence Small-Angle X-ray Scattering (GISAXS) (a-c) and in-plane GISAXS line cuts (d-f), q_y, of mesoporous films fabricated with the block copolymer PIB-*b*-PEO. Dashed lines correspond to nominal peak positions ratios (q/q*) 1, 1.9, and 3.

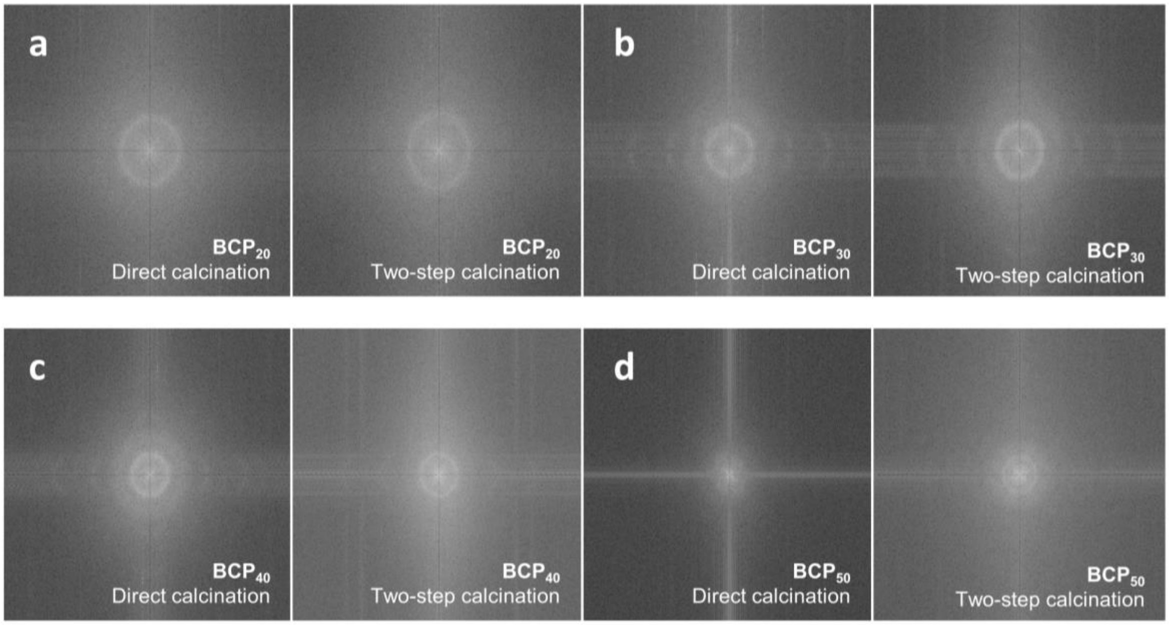

Figure S6. (a-d) 2D fast Fourier transform of the AFM images shown in Figure 7 of the main text.

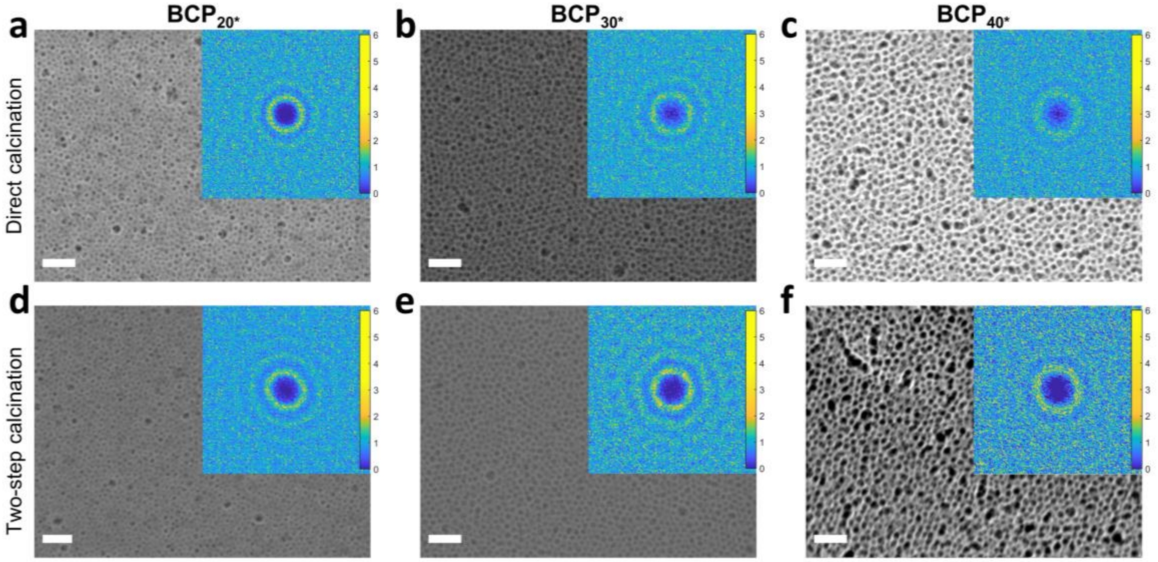

Figure S7. SEM images of mesoporous films fabricated with PIB-*b*-PEO after direct calcination in air (a-c) and after the two-step calcination (d-f) process. The inset corresponds to the 2D spatial distribution function calculated with the software CORDERLY to evaluate pore ordering. Scale bar: 100 nm.

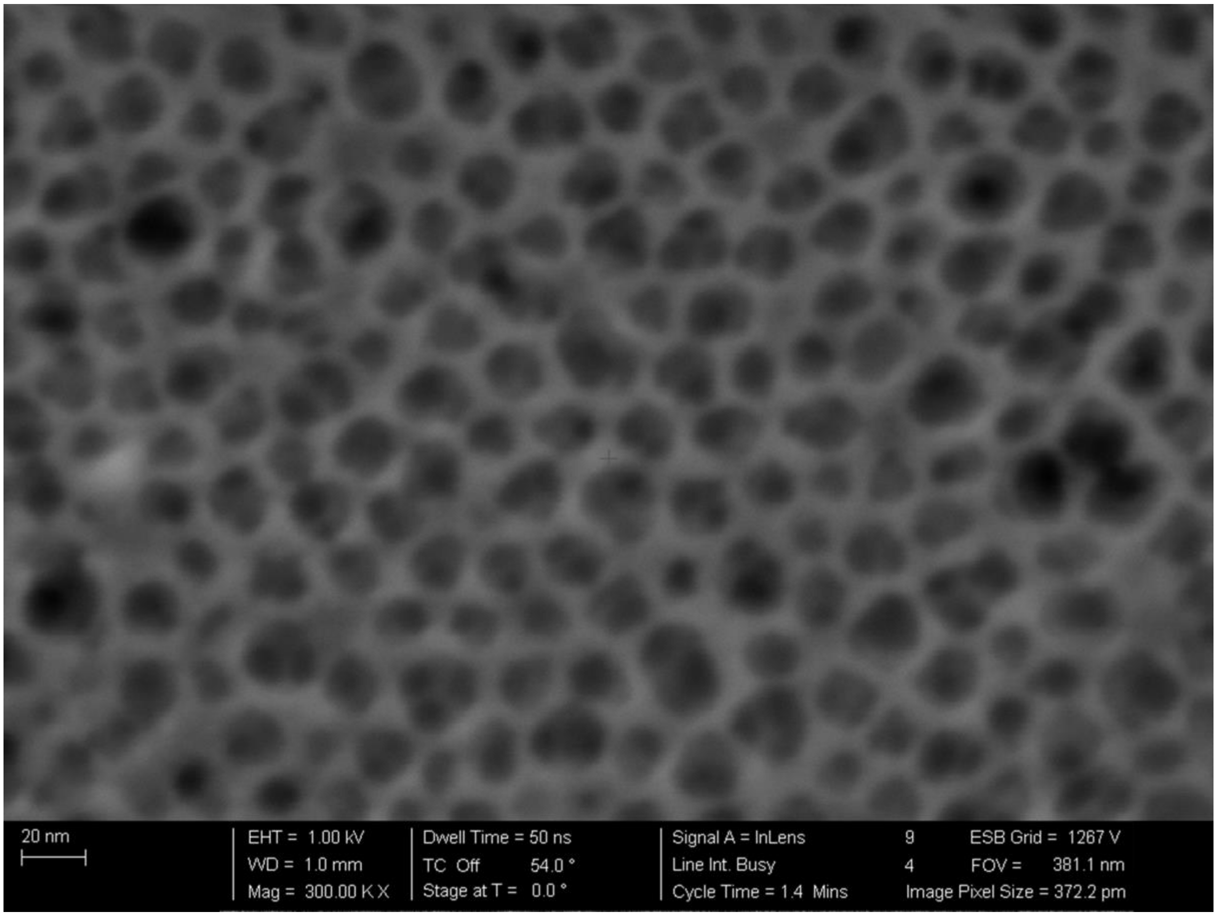

Figure S8. High magnification (300,000x) SEM image of the mesoporous surface of a BCP_{30} thin film fabricated with the block copolymer PIB-*b*-PEO after the two-step calcination process. At this magnification, it is possible to identify the interconnected nature of the mesopores.

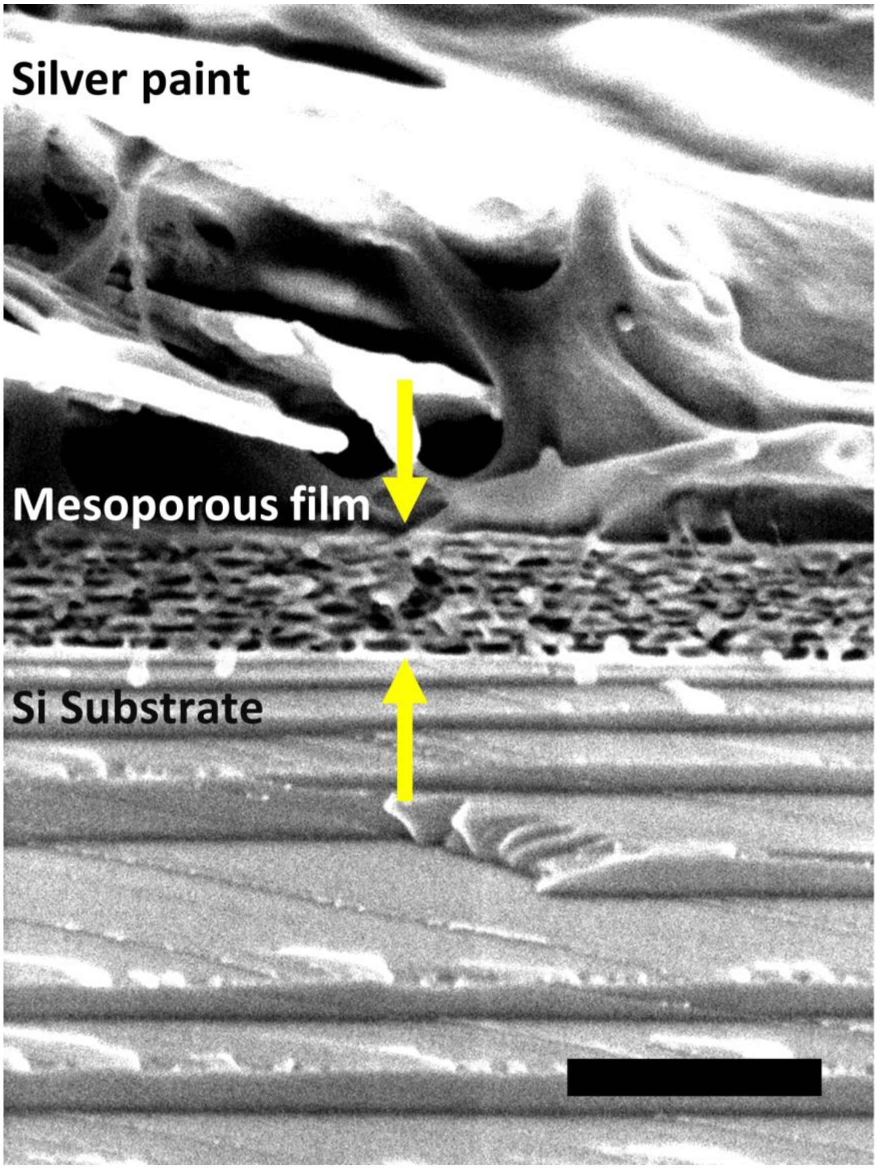

Figure S9. Cross-section SEM image of a two-step calcined thin film BCP_{50} PI-*b*-PEO. Scale bar: 300 nm.

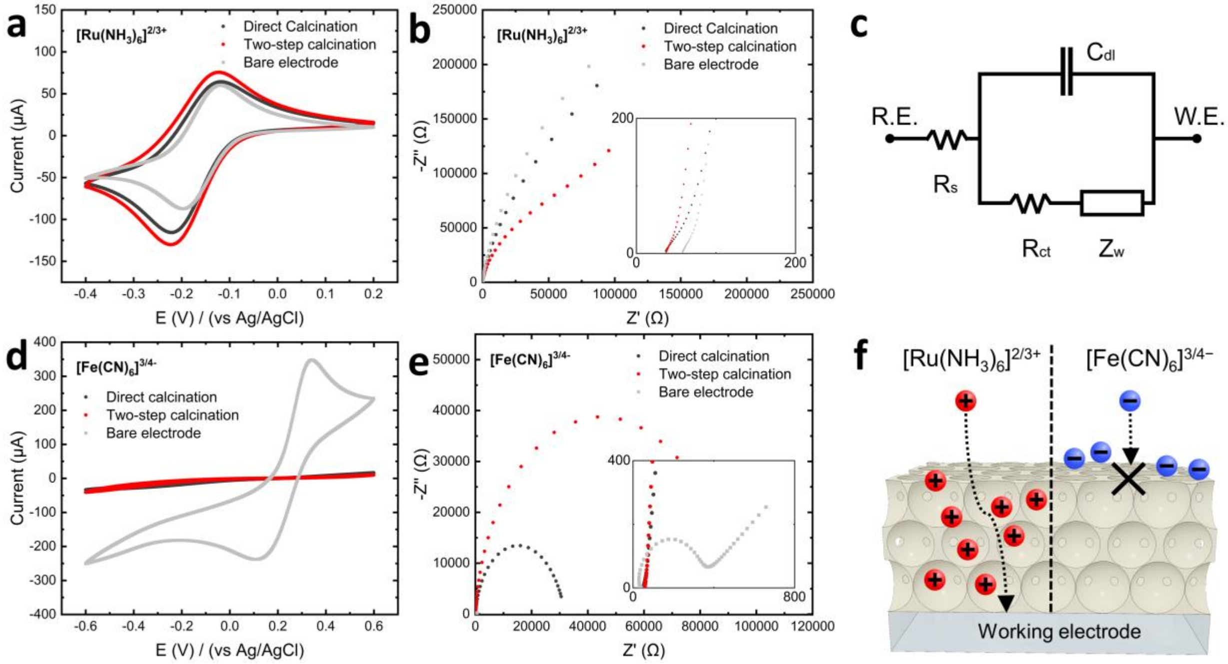

Figure S10. Electrochemical characterization of mesoporous aluminosilicate thin films fabricated with the 40% BCP PIB-*b*-PEO onto FTO coated glass (working electrode). Cyclic voltammogram (a,d) and Nyquist plot (b,e) measured using redox probes with opposite electric charge: (a,b) $[Ru(NH_3)_6]^{2+/3+}$ and (d,e) $[Fe(CN)_6]^{3-/4-}$ in PBS buffer. c) Randles circuit used to fit the Nyquist plots. F) Schematic of the permselective behavior observed on aluminosilicate mesoporous thin films, indicating the material has a negatively charged surface at pH 7.3.

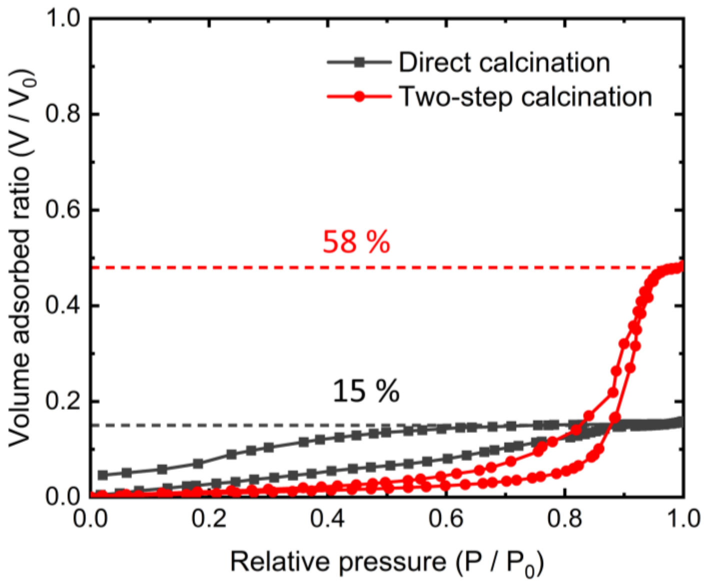

Figure S11. (a) Porosimetry measurements of the thin films BCP_{40} used for the enzyme storage experiment in QCM.
